# Pneumococcal Serotype Identification by Capsular Sequence Typing (CST): A Modified Novel Approach for Serotyping Directly in Clinical Samples

**DOI:** 10.3390/diagnostics11122353

**Published:** 2021-12-14

**Authors:** Nektarios Marmaras, Athanasia Xirogianni, Anastasia Papandreou, Efthymia Petinaki, Vana Papaevangelou, Maria Tsolia, Georgina Tzanakaki

**Affiliations:** 1 National Meningitis Reference Laboratory, Department of Public Health Policy, School of Public Health, University of West Attica, 115 21 Athens, Greece; marmarasn@hotmail.com (N.M.); axirogianni@uniwa.gr (A.X.); npapandreou@uniwa.gr (A.P.); 2 Department of Microbiology, Medical School, University of Thessaly, 411 10 Larissa, Greece; petinaki@med.uth.gr; 3 Third Department of Pediatrics, National and Kapodistrian University of Athens, University General Hospital “ATTIKON”, 124 62 Athens, Greece; vpapaev@gmail.com; 4 Second Department of Pediatrics, National and Kapodistrian University of Athens, “A&P Kyriakou” Children’s Hospital, 115 27 Athens, Greece; mtsolia@med.uoa.gr

**Keywords:** *S. pneumoniae*, non-culture serotyping, capsular sequence typing (CST)

## Abstract

As almost 60–70% of Invasive Pneumococcal Disease (IPD) is identified by nonculture methods in Greece, serotyping is of high importance for the better monitoring of pneumococcal serotypes due to the availability of conjugate vaccines. The aim of the study was the modification and direct application of the Capsular Sequence Typing (CST) assay in clinical samples in order to serotype *Streptococcus pneumoniae* culture-negative, Polymerase Chain Reaction (PCR_-positive samples, followed by CST group specific single-tube PCR assays. A two-step PCR modified assay was applied on a total of 306 samples (such as CSF, blood, pleural and middle ear fluids, isolates) obtained from 283 patients with IPD. The overall performance permits a rapid, accurate and cost-effective method for nonculture pneumococcal serotyping. As the management of IPD is closely related to the continuous monitoring of pneumococcal serotypes, the proposed approach proved to be a valuable tool for the typing and epidemiological monitoring of *S. pneumoniae*, for the evaluation of the overall impact of vaccination programs in the era of pneumococcal conjugate vaccines, in order to initiate the appropriate vaccination strategy.

## 1. Introduction

*Streptococcus pneumoniae* is a major cause of serious bacterial infections including meningitis, septicemia and pneumonia and is associated with significant morbidity and mortality worldwide. Young children and elderly people are at the highest risk, with a high incidence observed in those ages [1]. 

The ability of *S. pneumoniae* to cause disease is directly related to the capsule production, a polysaccharide structure external to the cell wall that provides resistance to phagocytosis and promotes the invasion of the host immune system by the bacteria [2]. To date, approximately 100 different pneumococcal serotypes have been identified, based on the unique antigen structure of the capsular polysaccharide, each with their own characteristics, adaptability for nasopharyngeal carriage and potential for invasive disease [3]. 

Over the years, in order to reduce the burden of Invasive Pneumococcal Disease (IPD), universal infant vaccination schemes have been initiated by initially introducing the 7-valent pneumococcal conjugate vaccine (PCV-7) in the early 2000s. An increase of non-PCV-7 serotypes observed in the following years [4,5] led to the replacement of PCV-7 in the routine infant immunization schedule. In the early 2010s, two vaccines, the 10-valent pneumococcal conjugate (PCV-10) and the 13-valent pneumococcal conjugate vaccines (PCV-13), offered a wider serotype coverage range [6]. The implementation of PCV universal vaccination has led to a significant decrease of IPD incidence among vaccinated children. 

However, as the vaccination is based on the pneumococcal capsular polysaccharides, immunization is expected to put further selective pressure on the pneumococcal population. Important vaccine effects following immunization are serotype replacement and capsule switch. As shown recently, serotypes that are not included in the aforementioned vaccines seem to emerge [7,8,9]. 

Consequently, when evaluating the potential impact of current or new vaccines, there is a need to predict the overall benefits, which are influenced by both declines in the disease incidence due to vaccine-targeted serotypes (VTs) and by an increase in the disease incidence due to non-vaccine targeted serotypes (NVTs). Hence, the management of IPD is closely related to the continuous monitoring of pneumococcal serotypes. 

*S. pneumoniae* serotyping is usually monitored by the serological determination of the capsular type by standard capsular tests after culture. The gold standard for serotyping is the Quellung or Neufeld test [10,11]. However, the method is time-consuming and expensive. On the other hand, several molecular serotyping methods are described by the use of sequential multiplex Polymerase Chain Reaction (PCR) assays [12,13,14,15,16]. However, due to the large number of pneumococcal serotypes used in order to assess the serotype, several multiplex PCRs are involved. Furthermore, many of the PCR-based serotyping assays, including Restriction Fragment Length Polymorphism (RFLP), Multi Locus Sequence Typing (MLST), DNA microarrays, Next Generation Sequencing (NGS) and Whole Genome Sequencing (WGS), require isolates in order to proceed [17]. 

On the other hand, Elberse et al. [18], in order to monitor the pneumococcal serotypes in the Netherlands, developed a molecular method, the Capsular Sequence Typing (CST) assay, for serotype identification on pneumococcal isolates. The aforementioned assay was based on the genotyping analysis of the capsular locus *wzh* gene and was achieved by the use of a mix of primers based on publicly available sequences of the capsular genes of known serotypes [19]. 

As almost 60–70% of *S. pneumoniae* cases are confirmed solely by PCR in Greece—mainly due to the early antibiotic treatment—there is a need for a diagnostic tool for direct serotype identification in culture-negative/PCR-positive biological fluids. 

The present study describes a modification of the CST assay in a two-step PCR protocol and its direct application on *S. pneumoniae* culture-negative/PCR-positive clinical samples (CSF, whole blood, pleural fluid, middle ear fluid), which were obtained from patients with IPD clinical manifestations such as meningitis and/or septicaemia, pneumonia, as well as otitis media and arthritis (synovial fluids), aiming for a nonculture pneumococcal serotype identification. Further, as the CST method occasionally identifies a group of 2–3 specific serotypes, individual PCR assays were developed and applied in order to define a single serotype.

## 2. Materials and Methods

### 2.1. Source of Specimens

A total of 306 samples identified as *S. pneumoniae* were obtained from 283 patients; these included 239 culture-negative PCR-positive clinical samples (whole blood (*n* = 36), cerebrospinal fluid (CSF) (*n* = 118), pleural fluid (*n* = 65), synovial fluid (*n* = 1), middle ear fluid (*n* = 15), blood culture (*n* = 2), pus (*n* = 2)) and 67 pneumococcal isolates. The samples were sent during 2010–2020 from hospitals throughout the country as the routine identification service provided by to the National Meningitis Reference Laboratory (NMRL).

All bacterial isolates received had previously been identified and serotyped with compatible culture methods (α haemolysis, optochin test, Quellung reaction), while the clinical samples were identified as *S. pneumoniae* by the use of a multiplex PCR assay described previously [20].

### 2.2. DNA Isolation

DNA isolation from clinical samples was carried out by the use of the MagCore^®^ Genomic DNA Whole Blood Kit (MagCore HF 16 nucleic acid extraction system, RBC Bioscience, New Taipei City, Taiwan).

For pneumococcal isolates, DNA extraction was carried out from a 24 h blood agar culture, as described previously [20]. 

### 2.3. Capsular Sequence Typing (CST) 

CST was based on the previously published protocol by Elberse et al. [18]. In order to meet the needs of serotyping directly for clinical samples, the method was modified in a two-step PCR assay. Specifically, during the first step, primers without M-tails were used, while during the second step, the PCR products obtained from the first PCR assay were submitted to the second PCR assay with primers extended with M-tails (i.e., universal forward and reverse sequences added as adapters to the 5′ end upstream and downstream from the oligonucleotides) (Table 1).

During the first step PCR assay, 0.8 mM dNTPs (New England Biolabs, Ipswich, MA, USA), 0.48 U/reaction Phusion^®^-High Fidelity DNA Polymerase (New England Biolabs, Ipswich, MA, USA) and 1.1× HF buffer (New England Biolabs, Ipswich, MA, USA), 0.8 μL primer mix (10 μΜ/each primer) (VBC, Vienna, Austria) and 2 μL DNA template were mixed in a 20 μL total reaction volume, under the following conditions: 98 °C for 1 min as initial denaturation, 98 °C for 14 s, 58 °C for 35 s and 72 °C for 38 s for 14 cycles, 98 °C for 13 s, 57 °C for 34 s and 72 °C for 37 s for 14 cycles, 98 °C for 12 s, 56 °C for 32 s and 72 °C for 35 s for 14 cycles, and 72 °C for 5 min as a final extension step, in a RoboCycler (Stratagene, San Diego, CA, USA). Further, 12 μL of the PCR product were stained with 3 μL GelRed loading buffer (6× Gel loading dye, Biotium, Fremont, CA, USA) subjected to electrophoresis in 2.0% (*w*/*v*) agarose gel (Nippon Genetics, Tokyo, Japan) and visualized under ultraviolet fluorescence light. 

During the second step PCR protocol, 0.6 mM dNTPs (New England Biolabs, Ipswich, MA, USA), 0.5 U/reaction Phusion^®^-High Fidelity DNA Polymerase (New England Biolabs, Ipswich, MA, USA) and 0.9× HF buffer (New England Biolabs, Ipswich, MA, USA), 0.6 μL primer mix (10 μΜ/each primer) (VBC, Vienna, Austria) and 2 μL DNA template (first PCR product) were added in a 20 μL total reaction volume under the following conditions: 98 °C for 1 min as initial denaturation, 98 °C for 10 sec, 55 °C for 30 s and 72 °C for 20 s for 20 cycles (+0.5 °C/cycle), 98 °C for 10 sec, 66 °C for 30 s and 72 °C for 20 s for 22 cycles, and 72 °C for 5 min as a final extension step, in a PikoThermalCycler (Finnzymes /ThermoFisher Scientific, Waltham, MA, USA). Further, gel electrophoresis was carried out in 5 μL of the PCR product stained with 1 μL GelRed loading buffer (6× Gel loading dye, Biotium, Fremont, California, USA) in 2.0% (*w*/*v*) agarose gel (Nippon Genetics, Tokyo, Japan). PCR products were visualized under ultraviolet fluorescence light.

### 2.4. PCR Product Purification and Sequencing

PCR products were purified according to the PCR-clean-up protocol, NucleoSpin^®^ Gel and PCR Clean-up kit (Macherey–Nagel, Düren, Germany) in a 30 μL final elution volume. Further, in order to test the purification yield, 5 μL of the purified product was stained with 1 μL GelRed loading buffer (6× Gel loading dye, Biotium, Fremont, CA, USA). Purified products were subjected to electrophoresis in 2.0% (*w*/*v*) agarose gel (Nippon Genetics, Tokyo, Japan) and visualized under ultraviolet fluorescence light. The purified products were sent for sequencing.

### 2.5. Sequencing Analysis

Chromatograms were analyzed by the use of Chromas software (http://technelysium.com.au/wp/chromas/, version 2.6.6, Technelysium Pty Ltd, South Brisbane, Australia, free downloaded (accessed on 10 September 2021)). Nucleotide sequences derived from the two DNA chains were compared to each other with ClustalW (https://www.genome.jp/tools-bin/clustalw, Bioinformatics tools provided by GenomeNet, Kyoto University Bioinformatics Center, Kyoto, Japan, free online software (accessed on 10 September 2021).

The nucleotide sequences were imported in the *S. pneumoniae* CST Typing Tool (https://www.rivm.nl/mpf/typingtool/spn, version 0.0, National Institute for Public Health and the Environment, Ministry of Health, Welfare and Sport, Catharijnesingel, Utrecht, The Netherlands (accessed on 10 September 2021)) (free online database of the National Institute for Public Health and the Environment, RIVM, Netherlands), and the *S. pneumoniae* serotype was automatically assigned [18].

### 2.6. Additional PCR Protocols

For cases in which the CST Typing Tool assigned a group of 2–3 serotypes, for further identification of a single serotype, additional single tube-PCR assays that are currently available were deployed. 

The specific primers used are presented in Table 2. All PCR assays were carried out under the same PCR program and reagents’ concentration: 1× Kapa 2G Fast Multiplex mix (Kapa Biosystems, Cape Town, South Africa), 0.3 μΜ of each respected primer (Table 2) and 2 μL DNA template were mixed in a 20 μL total reaction volume, under the following conditions: 98 °C for 30 s as initial denaturation, 98 °C for 5 sec, 65 °C for 12 s (−0.2 °C/cycle) and 72 °C for 15 s for 11 cycles, 98 °C for 5 sec, 63 °C for 12 s and 72 °C for 15 s for 27 cycles, and 72 °C for 1 min as a final extension step, in a Piko Thermal Cycler (Finnzymes/Thermo Fisher Scientific, Waltham, MA, USA). PCR products were stained with 3 μL GelRed loading buffer (6× Gel loading dye, Biotium, Fremont, CA, USA), subjected to electrophoresis in 2.0% (*w*/*v*) agarose gel (Nippon Genetics, Tokyo, Japan) and visualized under ultraviolet fluorescence light. 

### 2.7. Specificity

The specificity of the CST method was assessed from the 67 pneumococcal isolates of the known serotype by the Quellung reaction sent by the hospitals.

## 3. Results

### Capsular Sequence Typing (CST) 

The CST was shown to be well-performed in all clinical samples (*n* = 239) (whole blood samples, CSF pleural/middle ear/synovial fluids, blood cultures) and bacterial isolates (*n* = 67) identified previously by PCR as *S. pneumoniae*. As the same serotype was identified in two or more samples obtained from the same patient, one serotype was considered per patient/case in the present study.

The specificity was estimated at 100% and evaluated from the 67 *S. pneumoniae* isolates for which the serotype was identified by the Quellung reaction (PPV 100% and NPV 100%). 

The CST successfully directly identified a single serotype (or a pair of serotypes within the same group) in 49.12% of the cases (139/283); namely, serotypes 3, 7A/F, 8, 9N/L, 10A, 10B, 10F/C, 14, 15A, 16F, 18A, 19A, 21, 23A, 23B, 31, 37 and 42 (Table 3).

For the remaining cases, for which CST assigned a group of two or three serotypes, the further application of an additional CST-assigned group specific single-tube PCR assay with the use of previously published primers pairs was deployed. According to the results, the CST-assigned group specific single-tube PCR assay successfully further identified a single serotype in 31.44% (89/283) of cases as follows: serotype 11A/D (previously CST-assigned as a group of serotypes 11A/D, 18F); serotype 12F (previously CST-assigned as 12F/B); serotype 22F (previously CST-assigned as 22A/F,15B/C); serotype 15B/C (previously CST-assigned as 15B/C,23F) and serotype 20 (previously CST-assigned as 20,13) (Table 4). 

Finally, for 55/283 (19.4%) of the remaining cases for which the serotypes were CST-assigned in the following specific groups, 17A/35B/C, 24F/40, 25A/F/38, 33A/F/35A, 34/17A, 35F/47F, there was no possibility for a single serotype to be identified due to a lack of PCR assays being currently available (Table 5).

## 4. Discussion

The bacteriological confirmation and serotype determination of clinically suspected IPD is important for a detailed epidemiological surveillance of pneumococcal infections. As has been recently shown, the incidence of serotypes responsible for IPD can change overtime [8]. Taking into consideration vaccine pressure and the development of future conjugate vaccines, the continuous monitoring of serotypes is important in the era of pneumococcal conjugate vaccines. 

The necessity to understand serotypes’ specific epidemiology and their association with the disease types, the difficulties which are currently faced in the management of pneumococcal disease and the adoption of preventive measures has led to further research on nonculture pneumococcal capsular typing techniques. As more than 60% of the *S. pneumoniae* cases are currently identified by molecular methods, the need for molecular identification and typing into clinical samples directly has been increasing in recent years. 

It is well known that the gold standard for serotyping is the Quellung or Neufeld test; however, the method is time-consuming and not cost-effective as the type, group and factor sera are expensive and require bacterial isolates. On the other hand, taking into account that approximately 100 different pneumococcal capsular types currently exist, the molecular approach by the use of sequential multiplex PCR assays [12,25] and real-time PCR methods [26,27,28,29], although it allows the serotype identification of culture-negative PCR-positive clinical samples at a high percentage (75–90%), still remains time-consuming and expensive. 

Hence, in order to be able to identify the prevalent serotypes, there is a need for less time-consuming, more cost-effective assays, especially during the post-PCV-13 era, during which serotype diversity has generally increased, with diverse serotypes such as 24F, 22F, 8, 15A and 33F becoming important in countries that use PCV-13 [30,31]. 

The present study demonstrates that the direct application of the CST in clinical samples identified all 239 nonculture clinical samples. The majority (80.56%) of cases assigned a single serotype, either solely by CST or in combination with single-tube PCR assays targeting for a specific CST group. The application of a two-step PCR protocol improved the yield of the PCR product, creating a sensitive tool when applied directly to clinical samples. As a result, a single serotype was identified directly by CST, without discrepancies in almost 50% of the cases, while the further application of the CST group specific single-tube PCR assay targeting a group of 2–3 serotypes yielded an additional 31.4% single serotypes. 

There was a 100% correlation with the results obtained by the use of traditional serotyping methods for the 67 culture-confirmed cases in which the serotype was identified. 

In comparison to serotype identification performed by either conventional methods (Quellung) or by the application of sequential multiplex PCR assays, the proposed methodology provides the typing of nonculture samples in a relatively inexpensive, rapid (results can be obtained within 24 h) and reliable way by the direct identification of a single serotype or identification of a group of two or three serotypes, which can be further identified by single-tube PCR assay. Further, the proposed methodology is rather conventional and can be easily established in laboratories that are equipped for DNA template preparation and PCR. Sequencing apparatus is not essential as it can be carried out by an external laboratory.

## 5. Conclusions

The application of the proposed methodology for direct pneumococcal serotyping to culture-negative, PCR-positive clinical samples is relatively inexpensive, rapid and reliable and could ameliorate pneumococcal serotype surveillance. Furthermore, it may facilitate the close monitoring of evolving serotypes and vaccine effectiveness, which is crucial for the evaluation of the overall impact of pneumococcal vaccination programs and for designing future vaccination strategies. 

## Figures and Tables

**Table 1:** Primers’ sequences used for the CST protocol.

	Primer	Primer Sequence	Publication
Primers used for the first PCR	CST_1F	CATTCGCATATCGTTTTTG	Modified from Elberse et al. [18] (without M-tails)
	CST_2F	CATTCTCACATTATTTTTGATGT	
	CST_3F	CATTCGCACATCGTCTTTG	
	CST_1R	CTGAGCTCTTTTTTTCATGA	
	CST_2R	GTGAACTCGTTTCTTCATGA	
	CST_3R	CCGAGCTCTCTTTTTCATAA	
	CST_4R	CCGAGCTCTCTTTTTCATGA	
Primers used for the second PCR	CST_01-M13F	GTAAAACGACGGCCAGCATTCGCATATCGTTTTTG	Elberse et al. [18]
	CST_02-M13F	GTAAAACGACGGCCAGCATTCTCACATTATTTTTGATGT	
	CST_03-M13F	GTAAAACGACGGCCAGCATTCGCACATCGTCTTTG	
	CST_01-M13R	CAGGAAACAGCTATGACCTGAGCTCTTTTTTTCATGA	
	CST_02-M13R	CAGGAAACAGCTATGACGTGAACTCGTTTCTTCATGA	
	CST_03-M13R	CAGGAAACAGCTATGACCCGAGCTCTCTTTTTCATAA	
	CST_04-M13R	CAGGAAACAGCTATGACCCGAGCTCTCTTTTTCATGA	
CST sequencing	M13F(-20)	GTAAAACGACGGCCAG	
	M13R	CAGGAAACAGCTATGAC	

**Table 2:** PCR primers used for the additional single tube PCR assays for the identification of a single serotype among the specific group of serotypes assigned by CST.

CST-Assigned Serotype	Additional PCR Assays			
	Serotype	Primer	Product (bp)	Publication
11A/D, 18F	11A/D	11A-F- GGACATGTTCAGGTGATTTCCCAATATAGTG	463 bp	Pai et al. [21]
		11A-R- GATTATGAGTGTAATTTATTCCAACTTCTCCC		
	18	18-F- GCATCTGTACAGTGTGCTAATTGGATTGAAG	354 bp	Brito et al. [12]
		18-R- CTTTAACATCTGACTTTTTCTGTTCCCAAC		
22A/F, 15B/C	22A/F	22A/F-F- GAGTAT AGC CAG ATTATGGCAGTT TTATTGTC	643 bp	Pai et al. [21]
		22A/F-R- CTCCAGCACTTGCGCTGGAAACAACAGACAAC		
	22F	22F-F- CTTGTCAAGTATGCTGAGGATTTG	82 bp	Velusamy et al. [22]
		22F-R- AGATTTCTCCTGGATATAATGCGAT		
	22A	22A-F- CCCAGGACAATCACAAGAACTA	84 bp	
		22A-R- TGATGCTTGGCCAAATTGGAG		
15B/C, 23F	15B/C	15B/C-F-TTGGAATTTTTTAATTAGTGGCTTACCTA	496 bp	Pai et al. [21]
		15B/C-R-CATCCGCTTATTAATTGAAGTAATCTGAACC		
	23F	23F-F- TGGTAGTGACAGCAACGA	177 bp	Lawrence et al. [23]
		23F-R- CAAAGGCTAATTCAGCATC		
12F/B	12F/44	12F/44-F- TTCGGAGGGTCCGATTATATTT	149 bp	Velusamy et al. [22]
		12F/44-R- CTTTGGTAATCCACTGTTCTGG		
20,13	20	20-F- GAG CAA GAG TTT TTC ACC TGA CAG CGA GAA G	514 bp	Pai et al. [21]
		20-R- CTA AAT TCC TGT AAT TTA GCT AAA ACT CTTATC		
	13	13-F- TACTAA GGTAAT CTCTGG AAATCGAAAGG	655 bp	Da Gloria Carvalho [24]
		13-R- CTCATGCATTTTAT AACCGCTTT TTG TTC		

**Table 3:** Pneumococcal single serotype identification by the application of CST.

CST-Assigned Serotype	Final Serotype Identified	Number of Cases
3	3	45
8	8	18
10A	10A	2
10B	10B	1
14	14	3
15A	15A	11
16F	16F	2
18A	18A	1
19A	19A	15
21	21	8
23A	23A	15
23B	23B	5
31	31	2
37	37	2
42	42	2
7A/F	7A/F	2
9N/L	9N/L	4
10F/C	10F/C	1
TOTAL		139 (49.12%)

**Table 4:** Pneumococcal serotype identification by the application of CST in combination with specific single-tube PCR assays.

CST-Assigned Serotype	Single PCR Assays	Final Serotype Identified		Number of Cases
11A/D, 18F	11A/D	11A/D		18
	18			
12F/B	12F/44	12F		14
15B/C, 23F	15B/C	15B/C		28
	23F			
20,13	20	20		7
	13			
22A/F, 15B/C	22A/F	22F		22
	22A			
	22F			
	15B/C			
	23F			
TOTAL			89 (31.44%)	

**Table 5:** Pneumococcal serotype identification by the application of CST assigning two or two serotypes.

CST-Assigned Serotype	Single PCR Assays	Final Serotype Identified	Number of Cases
24F/40	NA *	24F/40	18
25A/F, 38	NA	25A/F, 38	7
33A/F, 35A	NA	33A/F, 35A	3
34/17A	NA	34, 17A	5
35F/47F	NA	35F/47F	7
TOTAL			55 (19.4%)

* NA: Not Assigned; single-tube specific PCR assays not currently available.

## Data Availability

Not applicable.
